# Concussions in Collision Youth Sports

**DOI:** 10.15844/pedneurbriefs-30-1-2

**Published:** 2016-01

**Authors:** Kathleen A. Linzmeier, Cynthia R. LaBella

**Affiliations:** 1 Institute for Sports Medicine, Ann & Robert H. Lurie Children’s Hospital of Chicago, Chicago, IL

**Keywords:** Concussion, Traumatic Brain Injury, Sports Medicine

## Abstract

Investigators from the University of Pittsburg, University of Arkansas, Lake Erie College of Osteopathic Medicine, and Boston Children’s Hospital/Harvard Medical College researched the incidence of concussions in youth hockey in relation to age and activity setting.

Investigators from the University of Pittsburg, University of Arkansas, Lake Erie College of Osteopathic Medicine, and Boston Children’s Hospital/Harvard Medical College researched the incidence of concussions in youth hockey in relation to age and activity setting. They prospectively followed 397 ice hockey players, aged 12-18 years, from 31 teams, during 2 competitive seasons. Team representatives were trained to record athletic exposures (AEs) and identify suspected concussions. A concussion was defined as any mild closed head injury involving altered cognitive functioning, signs/symptoms, or loss of consciousness of no longer than 1 minute, after direct or indirect blow to the head. Thirty-seven concussions were diagnosed during 23,3069 AEs, 12,784 in practices, 10,585 in games. The combined concussion incidence rate (IR) for games and practices was 1.58 per 1000 AEs. The majority of concussions (N=26, 70.3%) occurred during games. All identified mechanisms of injury involved player-to-player contact. More than half involved secondary contact with the boards. Forty-three percent of mechanisms were attributed to illegal contact leading to a penalty. The authors conclude that concussion incidence rates in youth ice hockey are similar to those in other collision sports, and that younger players have higher rates than older players. [1]

COMMENTARY. Concussions are mild traumatic brain injuries (TBIs) that are a growing public health concern. Heightened awareness of concussions has led to a 29.1% increase in emergency department visits for TBI between 2006 and 2010, with over 2.5 million total visits in 2010 [2]. Particular concern has been raised about the safety of youths participating in collision sports such as football, rugby, and wrestling in which intentional physical collisions (e.g. tackling, takedowns) are a required component. Hockey is also considered a collision sport; however, the collisions are either incidental or strategic (body checking) and not required for participation. Concussion rate in boy’s ice hockey (5.4 per 10 000 AEs) is the second highest in collision sports, and only slightly lower than the rate in youth football (6.4 per 10 000 AEs). Previous research has demonstrated 30% of the concussions in boy’s ice hockey result from body checking [3]. While this study did not specify body checking as a mechanism, it is important to note that all concussions resulted from player-to-player contact and more than half involved secondary contact with the boards, both of which occur with body checking. Forty-three percent of the mechanisms of concussion in this study were additionally attributed to illegal contact, highlighting the need for improved enforcement of current rules. A recent study demonstrated that implementing a “no checking” rule in a Pee Wee hockey league decreased the rate of concussions and all injuries by nearly threefold (IRR 2.83 for concussions, IRR 2.97 for all injuries) [4]. Such rules targeting collision reduction are essential to injury prevention. The American Academy of Pediatrics Council on Sports Medicine recommends delaying introduction of body checking until age 15 with strict reinforcement of rules promoting player safety [5]. Adoption of additional rules, policies and educational interventions to decrease unsafe tactics may further reduce the incidence of collisions that have been shown to lead to concussions, especially for youth participating in competitive hockey.
